# Infant Growth Spurts in the Context of Perceived Insufficient Milk Supply

**DOI:** 10.3390/nu16213657

**Published:** 2024-10-27

**Authors:** Riccardo Davanzo, Maria Elisabetta Baldassarre

**Affiliations:** 1 Nutrition Research Centre, University of Insubria, 21100 Varese, Italy; riccardo.davanzo@gmail.com; 2 Department of Interdisciplinary Medicine, Neonatology and NICU Section, University “Aldo Moro”, 70124 Bari, Italy

## 1. Self-Reported Insufficient Milk Supply and Unsettled Baby Behaviors

Breastfeeding is the best source of nutrition for most babies and has been recognized as a means of optimizing health and wellbeing. Despite the WHO’s widely accepted recommendation to exclusively breastfeed for approximately the first 6 months, with continued breastfeeding suggested along with the introduction of appropriate complementary foods for up to 2 years of age or longer, fewer than half of all infants under 6 months of age are exclusively breastfed (the WHO, https://www.who.int/health-topics/breastfeeding#tab=tab_1) (accessed on 1 September 2024).

The Lancet Breastfeeding Series aptly addressed this issue [1], identifying the maternal perception of inadequate breast-milk supply as a major reason for the introduction of commercial milk formulas and the untimely discontinuation of breastfeeding. This is particularly the case when maternal self-efficacy is undermined by the aggressive marketing of breast-milk substitutes. Unfortunately, health professionals on their part may misconceive a wide set of normal baby behaviors as reliable sign of feeding problems, without being able to provide appropriate advice and effective support to mothers.

In a systematic review of 120 studies, infant crying, unsatisfied hunger, fussiness, and short intervals between feeding times have been documented as commonly and hastily attributed to inadequate milk supply [2]. In order to provide an easy-to-understand explication for these, unsettled infant behaviors have been connected to the so-called infant growth spurt.2.

## 2. The Popularity and Inconsistency of Infant Growth Spurts

Growth spurts are short periods of time when a child shows a faster growth rate in height and weight until reaching physical maturity. From birth to 2 years of life, there is rapid—although not steady—growth [3], particularly in length/height, with concurrent and progressive reduction seen in growth velocity. Subsequently, the rate of growth continues to increase at regular intervals until puberty, when a true growth peak is easily recognized [4].

In addition to this consolidated body of knowledge, some credit can be attributed to the existence of growth spurts even in the first year of life, particularly in order to explain why many breastfed infants during certain periods suddenly want to suck more often and for longer (cluster feeding), placing the mother under stress and making necessary an interpretation of their behavior.

Some health care institutions recognize a periodicity to infant growth spurts, placing them at fixed times—2–3 weeks, 6 weeks, and 3 and 6 months [5]. Moreover, the term “infant growth spurt” has been embraced by websites on maternal health and/or breastfeeding [6,7,8], magazines for new parents [9], and certain public health recommendations on early childhood feeding [10].

A PubMed search restricted to studies on humans that were published in English and indexed up to 5 August 2024 with the keywords “Breastfeeding” AND “Growth Spurt”, followed by “Infant” AND “Growth Spurt” identified 21 and 469 articles, respectively, on the topic. After title and abstract screening, only ten articles could be considered pertinent to the issue, and, ultimately, six were judged to be relevant [11,12,13,14,15,16]. Thus, although the concept of infant growth spurts seems to be popular particularly in the domain of breastfeeding promotion, the published literature on this topic is definitively scarce and unsubstantial.

Greco et al. described non-linear weight growth in the first year of life among low-birth-weight infants [11]. However, this growth pattern, defined as “pulsatile” by the authors, was shown to become regular when weight checks were carried out every 2 weeks instead of every 3 days. In other studies, Lampl et al. serially recorded infant length, highlighting a so-called “saltatory” growth pattern, comprising phases of increase and stasis [13]. Nevertheless, neither Greco’s nor Lampl’s studies succeeded in identifying significant periodicity or an approximate age for these infant growth spurts.

It is a common experience that a breastfed baby can show irregular growth during the first months of life as a result of difficulties with breastfeeding or simply as a result of a temporary imbalance between the production of breast milk and the nutritional needs of the child. However, there is no evidence that this disproportion might be due to a biological trigger, as suggested by the term “spurt”, but rather simply to physiological variable maternal production, which is expected to be periodically and transiently reduced or frankly inadequate, even in a mother who successfully breastfeeds. Low milk production, insofar as the infant is healthy and properly latches to the breast, can be overcome in most cases by exploiting the mechanism whereby the greater the baby’s request and the longer time spent sucking at the breast, the greater the stimulus to produce breast milk [17]. In fact, the weight growth of a healthy breastfed infant may show, at subsequent checks, a slowdown or arrest of growth followed by phases of true weight recovery (short-term catch-up growth) rather than acceleration (growth spurts) triggered by an endogenous mechanism.

Consistently, renowned textbooks of Physiology [18] and/or Pediatrics [19] poorly recognize growth spurts in the first year of life as an auxological phenomenon. Additionally, the Growth Charts recommended by the WHO for monitoring children under the age of 2 do not suggest any increase in growth velocity for this age group [20].

## 3. Basic References to the Physiology of Lactation

Therefore, it is clear how and why the concept of infant growth spurts has gained popularity, although it is not clear to what extent this concept has been mythologized or represents a conscious oversimplification, which health professionals are ready to propose to a mother in lactational crisis. After all, it is still professionally preferable to clarify to the parents of a breastfed infant that changes in the infant’s feeding behavior, with phases of weight slowdown and recovery, depend on the physiology of lactation [21] rather than on an endogenous chrono-biological driver.

Helping mothers to improve their breastfeeding self-efficacy is crucial in order to avoid premature complementary feeding. Nevertheless, skilled counseling and support by health workers should be conceptually evidence based. Ultimately, it is important to clarify that growth spurts in the first year of life seem to be inadequately supported by current scientific knowledge, and have to be considered as a simple compelling theory.

## Data Availability

Not applicable.

## Abbreviations

UNICEF	United Nations International Children’s Emergency Fund
